# Efficacy of Pulsed Radiofrequency to Cervical Nerve Root for Postherpetic Neuralgia in Upper Extremity

**DOI:** 10.3389/fnins.2020.00377

**Published:** 2020-04-21

**Authors:** Yuanyuan Ding, Hongxi Li, Tao Hong, Peng Yao

**Affiliations:** Department of Pain Management, Shengjing Hospital of China Medical University, Shenyang, China

**Keywords:** postherpetic neuralgia, upper extremity, neuromodulation, cervical nerve root, pulsed radiofrequency

## Abstract

**Background:**

Postherpetic neuralgia (PHN) seriously affects a patient’s quality of life, and it is urgent to find a method that can effectively alleviate the PHN of the upper extremity.

**Objective:**

To observe the Efficacy of pulsed radiofrequency (PRF) to cervical nerve root for PHN in upper extremity under CT guidance.

**Study Design:**

Retrospective comparative study.

**Setting:**

Shengjing Hospital of China Medical University.

**Methods:**

Fifty patients with PHN in upper extremity were enrolled in Pain Management. Patients were randomized into two groups: cervical nerve root block (A group, *n* = 25) and cervical nerve root PRF (B group, *n* = 25). At each observation time, the general characteristics, visual analog scale (VAS), quality of life scores assessment (SF-36), the total efficacy rate, dosage of antiepileptic and narcotic analgesics, and the incidence of complications were followed up.

**Results:**

Compared with the preoperative, the postoperative VAS decreased, the physical component summary (PCS) and the mental component summary (MCS) increased in both groups (*P* < 0.05). The differences between group B and group A were statistically significant after 1 month, which could be maintained for 1 year (*P* < 0.05). The total efficacy rate of group A and group B was 52.0% and 80.0% at 1 Year, respectively. The total efficacy rate of group B was higher than that of group A (*P* < 0.05). The dosage of antiepileptic and narcotic analgesics in group B decreased significantly, and the decline was significant compared with group A (*P* < 0.05). The incidence of complications between the two groups were similar (*P* > 0.05).

**Conclusion:**

CT-guided PRF to cervical nerve root for the treatment of PHN in the upper extremity is safe and effective. PRF can replicate the location of pain, precise positioning, reduce trauma, and increased pain relief rate.

## Introduction

Postherpetic neuralgia (PHN) refers to pain in the lesion area after 3 months of herpes zoster. PHN is the most common complication of herpes zoster and is a common clinical disease in the pain department. Herpes zoster on the extremity and face is a high-risk factor for PHN due to the sensitivity of the affected area (Nalamachu and Morley-Forster, 2012). The PHN of the upper extremity often presents hyperalgesia or allodynia such as tactile pain, burning pain, tingling, numbness, etc., and often manifests as moderate to severe pain. This seriously affects a patient’s quality of life, and it is urgent to find a treatment that can effectively alleviate the PHN of the upper extremity.

Currently, PHN lacks effective treatment methods. At present, the commonly used treatment methods are medicines, physiotherapy, nerve block, etc. Among them, medicines are the most basic and most commonly used. Medication is mainly to promote nerve repair, adjust nerve function and symptomatic analgesia. However, PHN often maintains a long and severe pain, long-term use of antiepileptic or non-steroidal analgesics have a high incidence of adverse reactions; repeated corticosteroid injection increases the risk in older patients with more side effects. Therefore, patients are often discontinued because of intolerance. Early minimally invasive interventional therapy can significantly improve PHN, including pulsed radiofrequency (PRF) and radiofrequency thermocoagulation (RFT). Due to the special position, the upper extremity PHN needs to maintain the motor function and could not be treated with RFT. Therefore, neuromodulation therapy can be taken. At present, neuromodulation can use a stellate ganglion block, but the effect is slow, the maintenance time is short, and repeated treatment is needed.

Pulsed radiofrequency is a short intermittent pulse with 480 ms heat dissipation interval so that the temperature acting on the tissue does not exceed 42°C. It does not cause nerve damage (Choi et al., 2014), only plays a role in neuromodulation (Lee et al., 2015). Therefore, PRF can act on the cervical nerve root for upper extremity PHN.

In this article, the PRF was applied to the cervical nerve root for the upper extremity PHN, and its efficacy and satisfaction were comprehensively evaluated.

## Materials and Methods

### General Information

**FIGURE 1:**
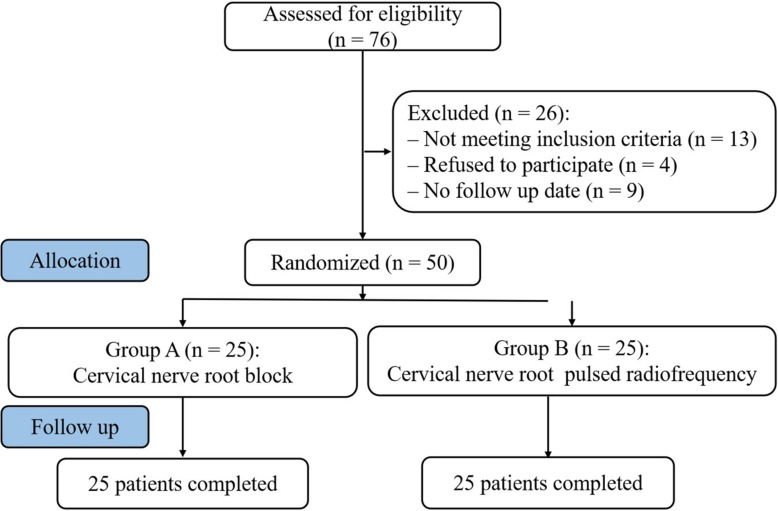
Study flowchart. All 50 patients were included in the treatment.

Fifty patients diagnosed with PHN in upper extremity from January 2018 to August 2018 were selected in the Department of Pain Management, Shengjing Hospital of China Medical University (Figure 1). Postherpetic pigmentations or lesions distributions were unilateral in all patients. Patients were randomly assigned to two groups according to the order of enrollment: cervical nerve root block (A group, *n* = 25) and cervical nerve root PRF (B group, *n* = 25). Both groups were injected with steroid drugs, supplemented with antiepileptic analgesics, narcotic analgesics, and neurotrophic drugs. The study was approved by the Ethics Committee of Shengjing Hospital, China Medical University. All patients were informed of the risks and complications before the operation, and written informed consents were obtained.

Inclusion criteria: (1) moderate to severe pain, visual analog scale (VAS) ≥ 5 points before enrollment; (2) upper extremity lesions healed, but persistent severe intractable pain, hyperalgesia, numbness, and paresthesia; (3) duration of PHN > 3 months; 4) age > 30 years old.

Exclusion criteria: local puncture area infection; mental illness, mental disorder, or disturbance of consciousness could not cooperate with the treatment; abnormal coagulation, pregnancy, or drug abuse history; severe liver and kidney dysfunction or severe cardiopulmonary disease.

### Surgical Operation

Under CT guidance, the patient was placed in the lateral position, and the affected side was on the upper side. The electrode plate of the radiofrequency was attached to the shoulder of the same affected side. The vital signs were monitored during the operation. CT scan was performed to locate the nerve root dominating region of the brachial plexus (BP) in the affected area of the rash. According to the CT scan, the needle path and the needle puncture point were determined. Conventional disinfection drape and local anesthesia with 0.5% lidocaine at the puncture point, radiofrequency needle punctured according to the path and angle. Under the guidance of the CT scan, the needle was gradually inserted until the tip reached the target position or the patient complained of abnormal sensation. The needle was stopped and aspirated to make no blood or CSF appear. Group A was treated with cervical nerve root block and group B with cervical nerve root PRF. The RF instrument (Baylis Medical Inc., Montreal, QC, Canada) was connected and tested: (1) 50 Hz, 0.1–0.3 V tested sensation, induced and duplicated the allodynia in the upper extremity herpes area; (2) 2 Hz, 0.1 V tested motor function, induced the upper extremity muscle spasm. After the adjustment position was satisfactory, 42°C PRF (2 HZ, 20 ms) was performed for 300 s. 2 ml of compound analgesic solution (2% lidocaine 2.5 ml + vitamin B12 0.5 mg + compound betamethasone 5 mg + normal saline 0.5 ml) was administered in B group. A group only administered the same dose of compound analgesic solution. Pull out the RF electrode needle and apply the gauze at the puncture site.

### Observations and Follow-up

Preoperative data, including gender, age, pain duration, pain location, preoperative VAS, and dosage antiepileptic and narcotic analgesics were recorded. Follow-up assessments were performed at 1 week, 1 month, 3 months, 6 months, and 1 year after operation, respectively. Patients were evaluated at follow-up visits by non-surgical medical staff.

(1) Visual Analog Scale (VAS): To assess the degree of pain, painless (0 points) to severe pain (10 points).
(2) Quality of life scores assessment (SF-36) (Lam et al., 2005): To assess the quality of life, including physical status and mental status. Physical state included: physical function, role physical, bodily pain, and general health; Mental state included: vitality, social function, role emotional, and mental health. The physical component and mental component were summarized and calculated.
(3) Total efficacy rate: Subjective symptoms and clinical signs were evaluated at 1 year. The efficacy rate was divided into three grades: excellent, effective, and ineffective. Excellent – pain, numbness and hyperalgesia disappeared; effective – pain and numbness were relieved; and ineffective – no improvement in symptoms. The total efficacy rate (%) = [(excellent + effective)/*n*] × 100%.
(4) Dosage of antiepileptic and narcotic analgesics: the antiepileptic analgesics included carbamazepine, pregabalin, and gabapentin; the narcotic analgesics was oxycontin.
(5) The incidence of complications: including local hematoma, nausea and vomiting, infection, headache, pneumothorax, physical activity abnormalities, and disorders.

### Statistical Analysis

The data were analyzed and processed by SPSS18.0 analysis software. The Kolmogorov–Smirnov test was used to assess the normality of measurement data. The variables with normal distribution were expressed as mean ± standard deviation (x ± SD). The values were compared using one-way analysis of variance, and LSD was used for pairwise comparison. The variables that did not conform to the normal distribution were expressed as the median (interquartile range). The values were compared using the Kruskal–Wallis test. Counting data were analyzed using Chi-square or Fisher’s exact test. *P* values < 0.05 were considered statistically significant.

## Results

### Patients Characteristics

The preoperative patient characteristics in group A and group B were compared. There were no significant differences in gender, age, pain duration, pain location, preoperative VAS, and the dosage of antiepileptic and narcotic analgesics (*P* > 0.05) (Table 1).

**TABLE 1:** Preoperation patients’ characteristics in A and B group.

Parameters	Group	
	A	B
Patients (n)	25	25
Gender (F/M,%)	13 (52.0%)/12 (48.0%)	14 (56.0%)/11 (44.0%)
Age (years, range)	55.26 ± 8.47 (42–68)	54.35 ± 8.32 (44–67)
Preoperation pain duration (M, range)	8.67 ± 4.23 (3–16)	8.58 ± 4.34 (4–17)
Pain side (n,%)		
Right	15 (60.0%)	16 (64.0%)
Left	10 (40.0%)	9 (36.0%)
Preoperation VAS	7.65 ± 1.43	7.47 ± 1.52
Preoperation analgesics dosage		
Carbamazepine (mg/d, n)	536.15 ± 76.24 (5)	542.21 ± 77.12 (6)
Gabapentin (g/d, n)	2.31 ± 0.42 (12)	2.29 ± 0.54 (11)
Pregabalin (mg/d, n)	417.48 ± 69.38 (8)	421.51 ± 71.45 (8)
Preoperation narcotic analgesics dosage		
Oxycontin (mg/day, n)	43.65 ± 11.34 (25)	43.79 ± 10.71 (25)

*A = cervical nerve root block, B = cervical nerve root pulse radiofrequency, VAS, visual analog scale. Data are presented as numbers (%) of patients or mean ± SD.*

### Intraoperative Conditions

**FIGURE 2:**
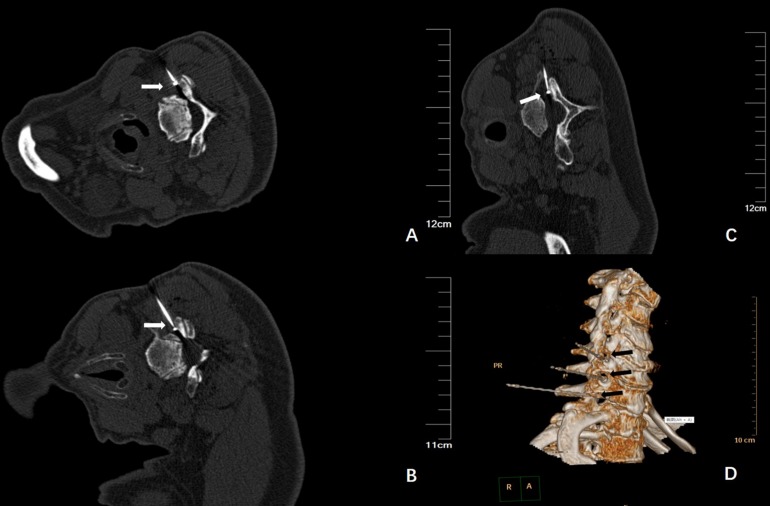
**(A)** Plain CT scan shows the needle tip was located at the right side of C5-6, as indicated by the arrow; **(B)** Plain CT scan shows the needle tip was located at the right side of C6-7, as indicated by the arrow; **(C)** Plain CT scan shows the needle tip was located at the right side of C7-T1, as indicated by the arrow; **(D)** The three-dimensional reconstruction shows the needle and the direction, located at the right side of the cervical nerve root, as indicated by the arrow.

The operation was successfully completed in all patients. The plain CT scan confirmed that the needle tip was located at the cervical nerve root C5-6, C6-7, and C7-T1 (Figures 2A–C); three-dimensional reconstruction was performed to further confirm the needle position (Figure 2D).

### VAS Pain Score

**FIGURE 3:**
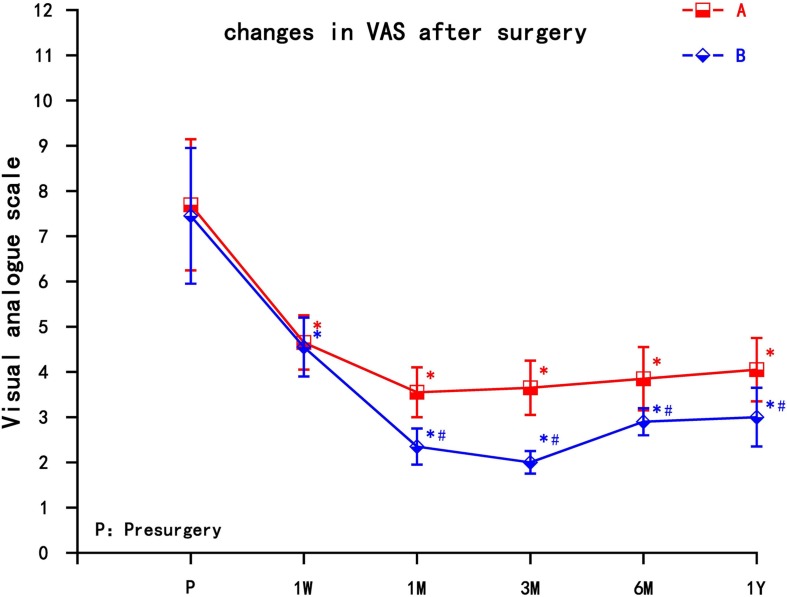
The comparison of VAS preoperation and postoperation in A and B group. A = cervical nerve root block, B = cervical nerve root pulse radiofrequency, Results are presented as means ± SD. *Compared with preoperation, *P* < 0.05; ^∗^compared with A group, *P* < 0.05.

Compared with the preoperative group, the postoperative VAS in group A and group B both decreased, and the difference was statistically significant (*P* < 0.05). The VAS of group A gradually increased after 1 month, while group B maintained long-term pain relief. The VAS decreased significantly in group A at 1 month, and then gradually increased, but still lower than preoperative; VAS decreased most significantly in group B at 3 months; the difference between group B and group A was statistically significant after 1 month, which could be maintained for 1 year (*P* < 0.05) (Figure 3).

### SF-36 Assessment

**FIGURE 4:**
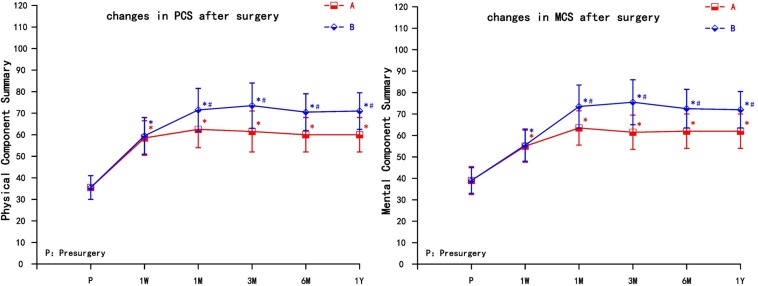
The comparison of quality of life scores (SF-36) preoperation and postoperation in A and B group. PCS, Physical Component Summary; MCS, Mental Component Summary; A = cervical nerve root block, B = cervical nerve root pulse radiofrequency; Results are presented as means ± SD. ^∗^Compared to preoperation, *P* < 0.05; ^#^Compared with A group, *P* < 0.05.

The two groups of patients obtained different degrees of improvement in the quality of life in the physical function, role physical, bodily pain, general health, vitality, social function, role emotional, and mental health after the pain relief. The physical component summary (PCS) and the mental component summary (MCS) increased in the two groups at each observation time point (1 week, 1 month, 3 months, 6 months, and 1 year) after the operation. Compared with preoperative scores, the difference was statistically significant (*P* < 0.05). The PCS and MCS increased significantly in group A at 1 month, then decreased gradually, but still higher than preoperative; the PCS and MCS increased most significantly in group B at 3 months; the difference between group B and group A was statistically significant after 1 month, which could be maintained for 1 year (*P* < 0.05) (Figure 4).

### Total Efficacy Rate

The total efficacy rate of group A and group B was 52.0% and 80.0% at 1 Year, respectively. The total efficacy rate of group B was higher than that of group A (*P* < 0.05) (Table 2).

**TABLE 2:** The comparison of total efficacy rate pre and postoperation in two groups (%).

Group	*N*	Excellent	Effective	Ineffective	The total efficacy(%)
A	25	8	5	12	52.0
B	25	12	8	5	80.0*

*Compared with A group, P < 0.05.

### Dosage of Antiepileptic and Narcotic Analgesics

**FIGURE 5:**
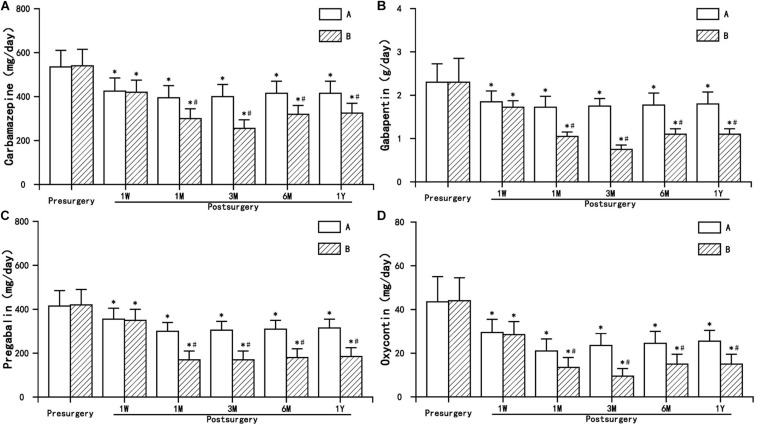
The comparison of the dosage of antiepileptic and narcotic analgesics preoperation and postoperation in A and B group. Results are presented as means ± SD. ^∗^Compared to preoperation, *P* < 0.05; ^#^compared with A group, *P* < 0.05. **(A)** Carbamazepine, **(B)** Gabapentin, **(C)** Pregabalin, and **(D)** Oxycontin.

The postoperative dosage of antiepileptic analgesics (carbamazepine, gabapentin, and pregabalin) and narcotic analgesics (Oxycontin) in the two groups decreased to varying degrees, and even discontinued, which was statistically significant compared to preoperation (*P* < 0.05). The dosage of antiepileptic and narcotic analgesics in group B decreased significantly, and the decline was significant compared with group A (*P* < 0.05) (Figure 5).

### Complications

During the hospitalization, the operations of the two groups were successfully completed. After the operation, both groups had local hematoma, nausea and vomiting, headache and other complications, but after local cold compress, symptomatic treatment and absolute supine, they recovered quickly without any serious adverse reactions. There were no other serious or permanent complications such as pneumothorax, infection, physical activity abnormalities and disorders in both groups. The incidence of complications between the two groups were similar (*P* > 0.05) (Table 3).

**TABLE 3:** The Complications in two groups(%).

Complications	Group	
	A	B
Local hematoma, n (%)	5 (20.0)	4 (16.0)
Nausea and vomiting, n (%)	1 (4.0)	0 (0.0)
Infection	0 (0.0)	0 (0.0)
Headache, n (%)	2 (8.0)	1 (4.0)
Dizziness, n (%)	2 (8.0)	2 (8.0)
Physical activity abnormalities and disorders, n (%)	0 (0.0)	0 (0.0)
Incidence of complications (%)	10 (40.0)	7 (28.0)

*A = cervical nerve root block, B = cervical nerve root pulsed radiofrequency.*

## Discussion

Postherpetic neuralgia is a chronic neuropathic pain that is characterized by spontaneous pain, hyperalgesia, and allodynia in the affected area. PHN usually persists and its pathogenesis and mechanism are unclear.

Herpes zoster is reactivated by the varicella-zoster virus that is latent in the body. The virus reaches the affected area along the descending sensory nerve, destroying the peripheral nerve tissues such as the dorsal root ganglia and the peripheral nerve, causing local tissue damage and inflammatory reaction, sensitizing peripheral nociceptors, and then developing into central sensitization, causing spontaneous pain and hyperalgesia (Feller et al., 2005). After the disappearance of the rash, the local skin still has pain and discomfort, lasting for more than 3 months is PHN. The occurrence of PHN is positively correlated with age and the location of herpes. The risk of PHN in upper extremity herpes is relatively high. Herpes in the upper extremity is more likely to develop into PHN, suggesting that the damage of sensitive nerve is more likely to lead to PHN than that of non-sensitive nerve and more difficult to treat (Nalamachu and Morley-Forster, 2012). PHN of the upper extremity is difficult to cure and complicated in etiology. Medicine treatment is often fast tolerated. In order to preserve the motor function of the upper extremity, it is impossible to use nerve damage treatment. At present, one of the better treatment methods is spinal cord stimulation (SCS). But the price is expensive, most patients can’t afford it, so it is difficult to use widely (Texakalidis et al., 2019).

Brachial plexus is composed of intercommunications among the ventral roots of the nerves C5-C8, and T1 (Acer and Turgut, 2019). BP innervates the sensation and movement of the upper extremity. Herpes zoster virus is latent in the dorsal root ganglia. Mostly the area of the upper extremity PHN is part of the BP. Therefore, the upper extremity PHN can be treated with cervical nerve root PRF. Podhajsky et al. (2005) found that PRF at 42°C caused reversible changes in the tissue, while RFT at 80°C produced destructive changes. After RFT at 80°C, the endoneurium appeared obvious edema, the dark-staining axoplasm (the early stage of Wallerian degeneration), and the structural destruction of the epineurium on the 2nd day. The extensive Wallerian degeneration appeared on the 7th day. The degenerated myelin lamina and vacuolar-filled axoplasm were seen, and the ruptured epineurium was also seen without nerve regeneration. After PRF at 42°C, endoneurium edema appeared on the 2nd day, no myelin or axonal pathological changes; edema disappeared at the 7th day, no further progress to obvious axonal injury, although there was still a thick endoneurium and epineurium collagen deposition (Podhajsky et al., 2005). The injury of the cervical nerve root can lead to dyskinesia of the upper extremity, so RFT cannot be used. The analgesic effect of PRF is not achieved by destroying nerves, and its mechanism is still unclear. Some studies have suggested that PRF interferes with the microstructure of nerve tissue, the generation of action potential and ectopic discharge, thereby reducing the excitability of neurons and blocking the transmission of pain information (Cosman and Cosman, 2005). PRF can induce early and long-term expression of the c-fos gene in the spinal dorsal horn (Higuchi et al., 2002; Van Zundert et al., 2005), and can reduce C fiber excitability by affecting ion channel function and ATP metabolism (Erdine et al., 2009). PRF can induce long-term inhibition of synaptic potential (Cahana et al., 2003), thereby inhibiting the transmission of pain. Therefore, PRF may block nerve conduction through neuromodulation (Abejón and Reig, 2003).

Pulsed radiofrequency is neuromodulation that exposes target neural tissue to electromagnetic fields generated by PRF and sends short bursts of high frequency current to relieve pain. The PRF is intermittent with a pulse frequency of 2 Hz and a current of 300–500 kHz. It is dispensed in a short interval of 20 ms and then intermittently for 480 ms so that the generated heat is diffused and the temperature produced does not exceed 42°C. Therefore, PRF only produces persistent neuromodulation and does not produce a destructive effect as RFT. Recent studies have found that PRF can produce neuromodulation and relieve trigeminal PHN (Ding et al., 2019). It has been found that PRF can inhibit the nociceptive-induced release of excitatory neurotransmitters (Huang et al., 2016), reduce the expression of calcitonin gene-related peptide (CGRP) in DRG (Ren et al., 2018), inhibit the expression of P2 × 3 receptor in DRG and spinal dorsal horn (Fu et al., 2019), and reduce the expression of peripheral of pro-inflammatory cytokines (TNF-α and IL-6) and β-Catenin in spinal cord (Vallejo et al., 2013; Jiang et al., 2019); At the same time, PRF can up-regulate GDNF transcription and translation (Jia et al., 2016; Hailong et al., 2018), up-regulate GABAB-R1, Na/K ATPase and 5-HT3r gene expression (Vallejo et al., 2013), increase histone acetylation and KCC2 expression by modifying KCC2 and partially restored GABA synaptic function (Liu et al., 2017). The effect of neuromodulation is slow, so cervical nerve root PRF combined with nerve block was used. Under the guidance of CT, the PRF to the cervical nerve root was more accurate, which can achieve rapid and long-lasting control of PHN avoiding the permanent damage of RFT to the nerve.

In conclusion, PRF cervical nerve root for the treatment of PHN in the upper extremity is safe and effective, can significantly alleviate the herpetic neuralgia of upper extremity, improve the quality of life in physical and mental, and reduce the dosage of antiepileptic and narcotic analgesics. PRF can replicate the location of pain, precise positioning, reduce trauma and increased pain relief rate. In the next step, we will conduct further research in a prospective study and gradually increase the number of patients later.

## Data Availability Statement

The datasets generated for this study are available on request to dingyy81@163.com.

## Ethics Statement

The studies involving human participants were reviewed and approved by the Ethics Committee of Shengjing Hospital, China Medical University. The patients/participants provided their written informed consent to participate in this study.

## Author Contributions

PY designed and conducted the study, including patient recruitment, data collection, and data analysis. HL collected the data. TH analyzed the data. YD prepared the manuscript draft. All authors approved the final manuscript.

## Conflict of Interest

The authors declare that the research was conducted in the absence of any commercial or financial relationships that could be construed as a potential conflict of interest.
